# Cytoskeletal Reorganization Drives Mesenchymal Condensation and Regulates Downstream Molecular Signaling

**DOI:** 10.1371/journal.pone.0134702

**Published:** 2015-08-03

**Authors:** Poulomi Ray, Susan C. Chapman

**Affiliations:** Department of Biological Sciences, Clemson University, Clemson, South Carolina, United States of America; University of Colorado, Boulder, UNITED STATES

## Abstract

Skeletal condensation occurs when specified mesenchyme cells self-organize over several days to form a distinctive cartilage template. Here, we determine how and when specified mesenchyme cells integrate mechanical and molecular information from their environment, forming cartilage condensations in the pharyngeal arches of chick embryos. By disrupting cytoskeletal reorganization, we demonstrate that dynamic cell shape changes drive condensation and modulate the response of the condensing cells to Fibroblast Growth Factor (FGF), Bone Morphogenetic Protein (BMP) and Transforming Growth Factor beta (TGF-β) signaling pathways. Rho Kinase (ROCK)-driven actomyosin contractions and Myosin II-generated differential cell cortex tension regulate these cell shape changes. Disruption of the condensation process inhibits the differentiation of the mesenchyme cells into chondrocytes, demonstrating that condensation regulates the fate of the mesenchyme cells. We also find that dorsal and ventral condensations undergo distinct cell shape changes. BMP signaling is instructive for dorsal condensation-specific cell shape changes. Moreover, condensations exhibit ventral characteristics in the absence of BMP signaling, suggesting that in the pharyngeal arches ventral morphology is the ground pattern. Overall, this study characterizes the interplay between cytoskeletal dynamics and molecular signaling in a self-organizing system during tissue morphogenesis.

## Introduction

A major objective in the skeletogenic field is to understand the sequential mechanisms that direct specification, condensation and overt differentiation during skeletal chondrogenesis. Local signaling from adjacent epithelia specify prechondrogenic fate in neural crest-derived mesenchyme, which then differentiate into chondrocytes several days later [1]. The critical intermediate step between specification and overt differentiation is condensation, which has two important features: firstly, mechanical forces control cell shape and organization, setting the characteristic size and shape of skeletal elements and concurrently modulate cell fate choice [2–5], and secondly, condensation is a prerequisite for overt chondrocyte differentiation [6]. However, the mechanisms that control these morphogenetic processes and the role of molecular signaling pathways in the pharyngeal arches are poorly understood. We proposed that the condensation process involves an inherently self-organizing system of specified mesenchymal cells, modulated by dynamic interactions between cells and their microenvironment. These interactions result in cell shape changes that organize the initially randomly oriented mesenchymal cell organization, apparent in progenitor populations, into an organized condensation.

The classic model of condensation, based mainly on studies of limb and trunk mesenchymal stem cell populations in micromass culture, involves aggregation and rounding up of cells, cell migration towards the center, cell proliferation and an inability of the cells to move away from the center [4, 7]. Remarkably few in vivo studies have examined the timing and condensation mechanism within the pharyngeal skeleton. A difficulty is that endochondral ossification of pharyngeal arch skeletal elements occurs over a six day period during chick development. The chick embryo, which becomes increasingly inaccessible beyond HH24 (Hamburger and Hamilton) [8], sinks beyond reach into the yolk and is enveloped in multiple membranes, which complicates in vivo analysis. Each step acts as a prerequisite for the sequence to move to the next phase: specification (epithelial mesenchymal interactions), condensation (cytoskeletal rearrangements), and chondrocytes (overt differentiation). Patterning cartilage in the correct position and of the correct size and shape is also dependent on this sequence. Moreover, micromass culture studies are not ideal for modeling in vivo mechanical forces. For example, the spot and stripe-like condensations observed in micromass cultures following seeding never occur in intact tissues [9, 10]. Indeed, cell streams in vivo migrate into the pharyngeal arches and therefore the aggregation of dissociated cells observed in micromass cultures is unrepresentative of the in vivo situation. Additionally, the 3D structure of surrounding tissues in vivo and the inherent mechanical forces in operation are not recapitulated. These challenges have prohibited functionally addressing some outstanding key questions. By creating an in toto explant system we were able to examine the interplay between spatiotemporal cell shape dynamics and molecular mechanisms in specified prechondrogenic mesenchyme.

We investigated three questions related to the nature of the condensation process, (1) the timing and nature of dynamic cytoskeletal re-organization in specified prechondrogenic cells; (2) the identity of the principle molecular signaling pathways during cytoskeletal reorganization; and (3) and the effect of cytoskeletal reorganization on downstream gene expression required for chondrocyte differentiation. Our focus in this study was restricted to the nature of the condensation process with regard to timing and molecular signaling, and therefore, we did not investigate the magnitude of the physical forces involved.

Our results demonstrate that ROCK and Myosin II driven actomyosin contractions and differential cell cortex tension within the prechondrogenic mesenchyme drives cytoskeletal rearrangements, and the resultant cell shape changes are a prerequisite for mesenchymal condensation. Cytoskeletal reorganization is responsible for activating downstream BMP and FGF signaling, while negatively regulating TGF-β signaling. We have further determined that BMP signaling is instructive in dorsalizing the proximal pharyngeal condensations, but does not influence the ability of the mesenchyme to condense. Disrupting actomyosin contraction driven cytoskeletal rearrangements alone was sufficient to prevent condensation, inhibit *SOX9* expression and prevent chondrocyte differentiation. Thus, our findings indicate that cytoskeletal rearrangement is required for condensation and subsequence overt chondrocyte differentiation, and furthermore, that these cell shape changes regulate the downstream activity of several signaling pathways.

## Materials and Methods

### Chick embryos

Clemson University IACUC approved the study, protocol number 2011-041. Fertilized chicken eggs were obtained from the Clemson University Poultry Farm and incubated at 38.5°C in a humidified incubator to the desired stage. The embryos were harvested in normal saline solution and staged according to the Hamburger-Hamilton table of normal stages [8].

### Slice cultures and inhibitor treatment

Slice cultures were performed at stages HH25 and HH28. Using a flame sharpened tungsten needle (0.125 mm, World Precision Instruments), a transverse slice of the head region was obtained by making two sharp cuts, rostral and caudal to the otic vesicle downward through the boundaries of the first/second and the second/third pharyngeal arches respectively. The tissue was then washed in L15 media containing 1% Penicillin and Streptomycin (Pen Strep), placed flat on a Millicell cell culture insert (PICMORG50, Millipore) and cultured in a humidified incubator with 5% CO_2_ at 37°C for 24 hours. Neurobasal media (21103–049, Gibco) containing B27 (17504–044, Gibco), supplemented with 5% Fetal Bovine Serum (FBS) (Fisher), 1% Pen Strep (15140–122, Gibco) and 1% Glutamax (35050–061, Gibco) was used for culturing the tissue. Media was exchanged every 12 hours. The inhibitors used were 170 μM Blebbistatin (B0560, Sigma), 50 μM Cytochalasin D (PHZ1063, Molecular Probes), 100 μM LDN193189/BMP receptor inhibitor (04-0074-02, Stemgent), 100 μM SU5402/FGF receptor inhibitor (Pfizer), 200 μM XAV939/Wnt pathway inhibitor (Sigma, X3004), 250 μM Y27632/ROCK inhibitor (Y0503, Sigma), 10 μg/ml Recombinant Human BMP4 (314-BP-010, R&D Systems). The control media contained equivalent amount of the respective solvents (DMSO or water).

### Tissue preparation for immunohistochemistry

Fixed tissue samples were washed twice in 1X Phosphate Buffered Saline (PBS) and then infiltrated with 5% and 15% sucrose/1X PBS solutions overnight [11]. Next, the tissues were washed in a 7.5% gelatin/15% sucrose solution for several hours at 40°C in a water bath and embedded in a cryomold using 2-Methyl Butane and dry ice. Tissues were cryosectioned at 40 μm using a Leica CM3050 cryostat.

### Cell shape visualization

Chick embryos/explants were fixed in 4% paraformaldehyde (PFA) for 12–18 hours at 4°C. Tissues were embedded and cryosectioned as described above. Cryosections were washed once in 1X PBS and 3 times in 1X PBT (1X PBS + 0.1% TritonX-100 + 1% BSA) for five minutes each. Next the sections were blocked in 1X PBT with 10% goat serum for one hour. Anti-beta catenin antibody (rabbit polyclonal, #ab6302, Abcam, RRID:AB_305407) diluted 1:100 in 1X PBT was applied for two hours at room temperature in a humidified chamber. The slides were washed several times in 1X PBS, followed by 1X PBT washes, each for five minutes. Alexa Fluor 568 Phalloidin (1:40 in 1X PBT) (Molecular Probes) and Goat anti-rabbit Alexa Fluor 488 secondary antibody (1:400 in 1X PBT) (Molecular Probes) were applied for two hours at room temperature in a humidified chamber followed by several 1X PBS and 1X PBT washes of five minutes each. The sections were then incubated with Hoechst 33342 (B2261, Sigma, 200 μg/ml 1X PBS) for 30 minutes. A final wash with 1X PBS for five minutes was followed by incubation in Equilibration buffer (Molecular Probes) for five minutes and mounting in Slowfade (S-2828, Molecular Probes) for imaging.

### Immunohistochemistry

Embryos and tissue explants were fixed in either 4% PFA or 10% Neutral Buffered Formalin (NBF) for one hour on ice. The samples were embedded and cryosectioned as described. The immunolabeling protocol was the same as the cell shape visualization protocol described above except that the incubation time with the primary antibodies ranged from overnight (pSmad2 and pERK) to four days (pSmad1/5/8) at 4°C. For pERK staining, antigen retrieval was performed by microwaving in Antigen Unmasking solution (H-3301, Vectorlabs). The following antibodies were used: primary antibodies used were pSmad2 (1:25, rabbit polyclonal, 3101, Cell Signaling Technology, RRID:AB_331673), pSmad1/5/8 (1:100, rabbit polyclonal, 9511, Cell Signaling Technology, RRID:AB_331671), pERK (1:5, rabbit polyclonal, 9101, Cell Signaling Technology, RRID:AB_2315113); and the secondary antibody was Rabbit Goat anti-rabbit Alexa-Fluor 488 secondary antibody (1:400, A-11008, Molecular Probes).

### In situ hybridization

Whole-mount and section in situ hybridization was performed as previously described [1].

### Imaging and image analysis

All imaging was performed using a Nikon TiE inverted confocal microscope with a Roper Scientific HQ2 camera. Image processing and analysis were performed using Nikon NIS Elements version AR 3.2. Adobe Photoshop was used to prepare composite images. Cell numbers were quantified by using ImageJ.

Quantification—In each case a standard 800x800 pixel region was quantified using the analyze particle function in ImageJ. For example, when quantifying pSmad2, the number of pSmad2 positive nuclei was divided by the total number of Hoechst stained nuclei and converted to a percentage. For quantification of cell density during columella and extracolumella condensation and overt differentiation, automated cell counting using the analyze particles function in Image J was performed. The 800x800 pixel region was converted to an 8-bit grayscale image followed by thresholding. In the case of extracolumella, due to the very high density of cells, the watershed (binary) function was additionally used. Lower and upper limits of the pixel size to be considered for counting were set so as to eliminate background noise and artifacts. For nuclear morphometric analysis, circularity and deformation index was calculated using Fiji (ImageJ) software. Nuclear deformation index is defined by (x-y)/(x+y), where x is the length of the major axis of a nucleus and y is the length of the minor axis of a nucleus. Statistical analysis was performed using t-test and one-way ANOVA (JMP software version 9 or 12). A p value of < 0.05 was considered significant.

## Results

**Fig 1:**
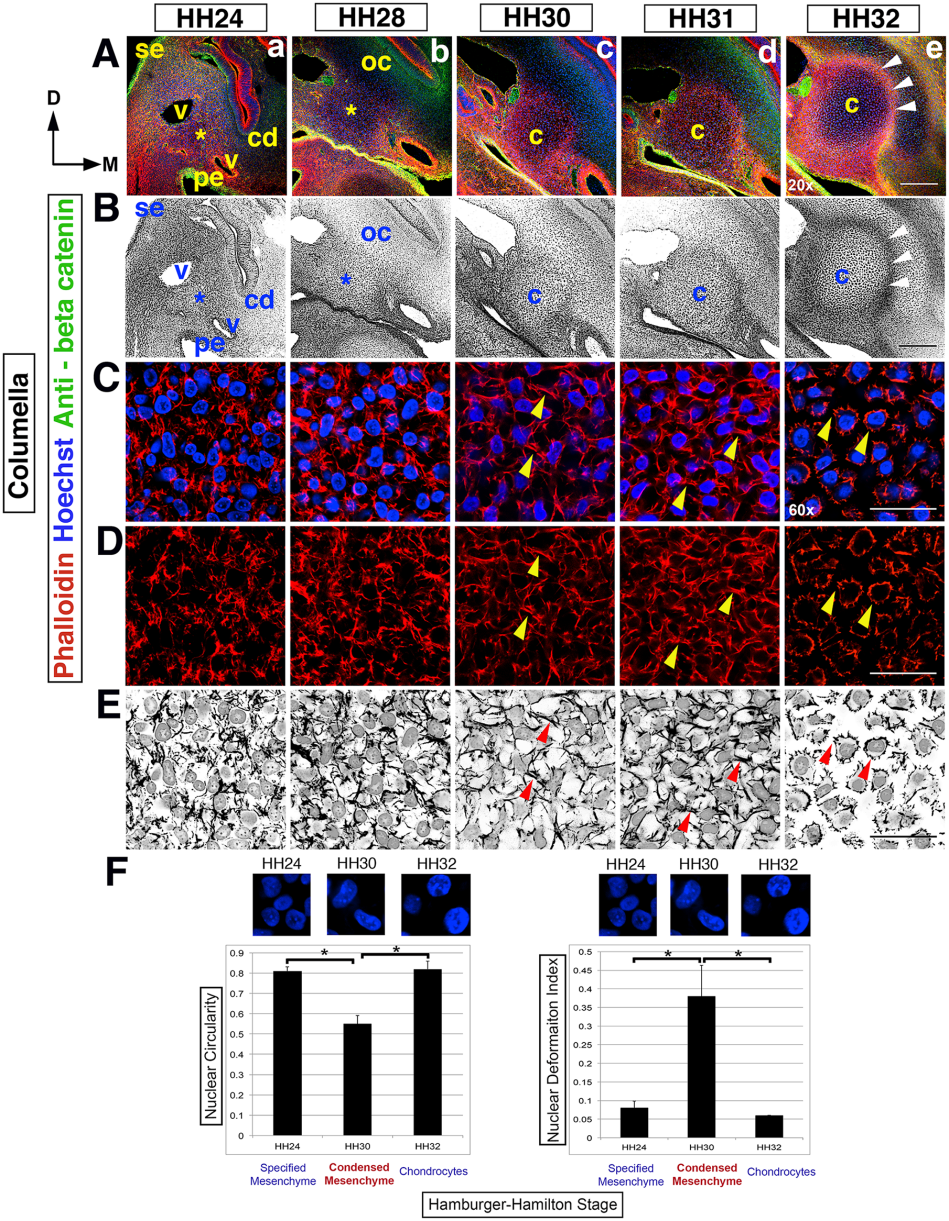
Dynamic cell shape changes reveal the timing of the columella. (Rows A,B) Overview of columella condensation in the context of surrounding tissues, from HH24-32 in transverse section at the 2^nd^ pharyngeal arch level, left side of the head (20x). Dorsal (D) is to the top, the midline (M) is to the right and surface ectoderm (se) is to the left. (A) Hoechst (blue)—nuclear DNA, Phalloidin (red)—F-actin, anti-beta catenin (green)—cell membranes and epithelia. (B) Black and white view. (Rows A,B) An asterisk indicates the position of nascent columella and the overlying nascent otic capsule (oc), from HH24-28. The nascent columella is surrounded by the cochlear duct (cd), pharyngeal endoderm (pe) and blood vessels (v). At HH30, the columella (c) condenses and by HH32 there is overt chondrocyte differentiation in the columella and overlying crescent shaped otic capsule. The perichondrium (arrowheads) surrounding the columella is apparent. (Rows C-E) F-actin rearrangements during columella condensation are apparent at higher magnification (60x). F-actin in red and nuclei in blue, with black and white images highlighting F-actin rearrangements and nuclei (row E). (Rows C-E) HH24-28, F-actin is disorganized and nuclei rounded in shape. At HH30/31, cell-cell actin bridges form (arrowheads), cells adopt rhomboid shapes and concomitantly distort nuclear shape. At HH32, overt differentiation is observed with cell-cell actin bridges replaced by cell-ECM adhesions leading to stellate shaped cells with rounded nuclei. (F) Quantification of nuclear circularity and deformation index during condensation and overt chondrogenesis. At HH24, nuclei are rounded with little distortion; in contrast, at HH30 during condensation, there is significant loss of nuclear circularity and a significant increase in the deformation index. This reverses during overt differentiation of chondrocytes when the nuclei of the now stellate shaped cells become rounded once more. Asterisk indicates p value <0.05. Abbreviations: cd-cochlear duct, c-columella, D-dorsal, M-midline, oc- otic capsule, pe-pharyngeal endoderm, se-surface ectoderm and v-blood vessel. Scale bars represent 150 μm (20x) and 25 μm (60x).

**Fig 2:**
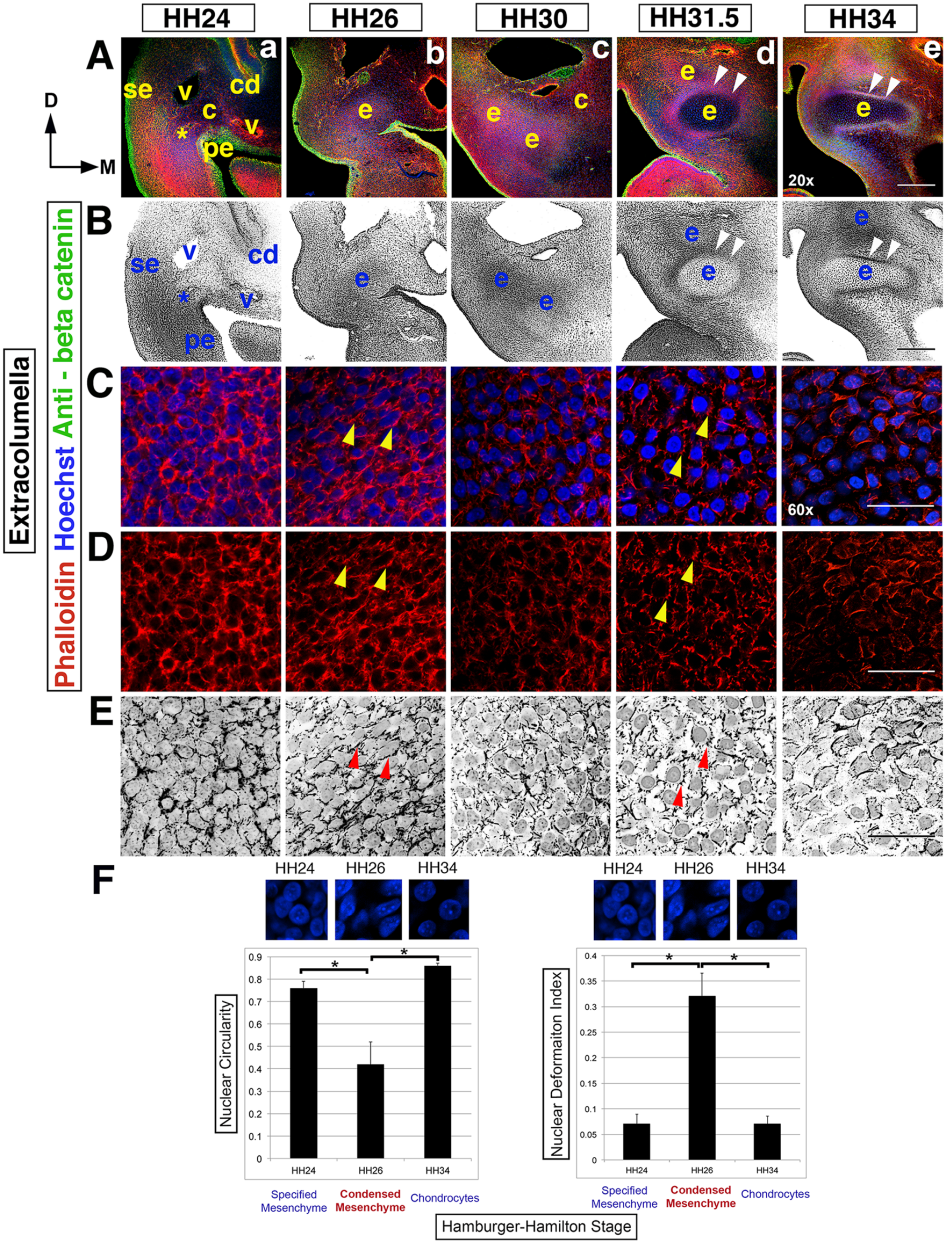
Dynamic cell shape changes reveal the timing of the extracolumella condensation. (Rows A,B) An overview of extracolumella condensation in the context of surrounding tissues, in transverse section at the 2^nd^ pharyngeal arch level, from HH24-34, left side of the head (20x). Dorsal (D) is to the top, the midline (M) is to the right and surface ectoderm (se) is to the left. (A) Hoechst (blue)—nuclear DNA, Phalloidin (red)—F-actin, anti-beta catenin (green)—cell membranes and epithelia. (B) Black and white view. (Rows A,B) At HH24 an asterisk indicates the position of the initially disorganized cells of the nascent extracolumella (e), which lies ventro-lateral to the nascent columella. At HH26, tissue organization and thus condensation, occurs in the extracolumella. By HH30 the extent of extracolumella formation is revealed, with the nascent perichondrium visible from HH31.5 (arrowheads). (Rows C-E) Dynamic F-actin rearrangements in the extracolumella region are apparent at higher magnification (60x). F-actin in red and nuclei in blue, with black and white images highlighting nuclei and F-actin (row E). (Rows C-E) At HH24 nascent extracolumella tissue is densely packed with disorganized F-actin bundles. At HH26, organized cell-cell connections (arrowheads) become apparent as cells undergo condensation. Cells are rhomboid shaped, with deformation of nuclei. By HH30, cell-cell F-actin is reducing. Next, cell-ECM adhesions, producing stellate shaped cells with rounded nuclei become predominant during the tissue stabilization phase. Overt differentiation of chondrocytes is apparent at HH34. (F) There is significant loss of nuclear circularity and an increase deformation index during condensation at HH26. Thereafter, cell-cell F-actin contacts are diminished and cell-ECM adhesions resulting in stellate shaped cells with rounded nuclei become predominant during overt differentiation. Asterisk indicates p value <0.05. Abbreviations: cd-cochlear duct, c-columella, D-dorsal, M-midline, exc-extracolumella, pe-pharyngeal endoderm, se-surface ectoderm and v-blood vessel. Scale bars represent 150 μm (20x) and 25 μm (60x).

The aim of the study was to elucidate mechanisms governing condensation during the multiday period between mesenchyme specification and overt chondrogenesis in the chick embryo (embryonic day E3-8/HH18-34). Local epithelial to mesenchymal interactions specify post-migratory neural crest-derived mesenchyme to a chondrogenic fate by HH18 [1]. When cultured in isolation, this specified mesenchyme self-organizes and condensation and chondrogenesis occur in a cell autonomous manner [1]. We hypothesize that dynamic cytoskeletal reorganization changes the shape of individual cells, which in turn, directs overall tissue organization. The significance of focusing on the Columella (2^nd^ arch) in our experimental paradigm is that several aspects of condensation could be investigated simultaneously. The columella and extracolumella elements are comprised of proximal (dorsal) and distal (ventral) mesenchyme cell populations with dual cell fates: replacement (ossified) cartilage and persistent cartilage, respectively (Figs 1 and 2) [12].

### F-actin organization and rhomboid cell shapes reveal the timing and nature of condensation

To determine the timing and physical nature of mesenchyme condensation, morphological progression of the nascent columella was analyzed using fluorescent immunohistochemistry on transverse head sections (Fig 1A–1F). At low magnification, early morphogenesis appears uneventful, exhibiting a disorganized mesenchyme. The nascent columella mesenchyme (asterisk) lies within the boundaries created by the lateral cardinal vein, the medial dorsal aorta, the second pharyngeal pouch endoderm in the ventral aspect, and dorsally by the otic capsule mesenchyme lateral to the cochlear epithelium (Fig 1Aa and 1Ba). The columella and future otic capsule have indistinguishable gross morphology at HH24, even though their cellular origin differs: mesoderm versus neural crest-derived mesenchyme, respectively.

Progressive cellular and tissue morphogenesis between HH24-32 is revealed at higher magnification (Fig 1C and 1D). Corresponding black and white images highlight nuclei shape, cortical actin organization and ECM between cells (Fig 1E). Rounded cell nuclei are arranged haphazardly at HH24 (Fig 1Ca). Movement of cells within this tissue is fluid, with continual rearrangement of cell shape as actin cycling occurs. The organization of the actin cytoskeleton is heterogeneous within the mesenchyme cells due to the irregular cortical distribution of F-actin fibers.

As the cytoskeletal components establish a new equilibrium, the previously irregular shaped cells and rounded nuclei adopt a stretched, rhomboid appearance (Fig 1Cc–1Ec, HH30). Actin bridges become apparent between cells (arrowheads) with overlapping branch-like intercellular processes resembling filopodia that form a web-like network of cell-cell connections. The establishment of homogeneity in the F-actin organization and rhomboid shaped cells with their filopodia-like cell-cell connections is concurrent with the formation of organized tissue in the columella region (Fig 1Cc,d–1Ec,d). The onset of tissue stabilization is observed at HH31 as the processes between cells begin to retract and cell shape becomes more ‘relaxed’, with nuclei recovering their rounded shape. At HH32, the intercellular processes have completely retracted and there is formation of multiple contact points with the ECM, which are likely to be focal adhesions. These changes contribute to the cell’s new ‘spiky’, stellate shape with rounded nuclei characteristic of overt differentiation into chondrocytes (arrowheads, Fig 1Ce–1Ee). The focal adhesions essentially spot-weld the cell membrane to the extracellular matrix, thereby promoting tissue stabilization [13, 14]. Furthermore, the increasing distance observed between cells results not only from the loss of cell-cell connections, but also increased deposition of ECM and changes in the composition and alignment of the ECM [15, 16].

Additionally at HH28, otic capsule progenitors begin to differ visually, appearing lighter than the columella mesenchyme (asterisk, Fig 1Bb). At HH30/31, the nascent perichondrium can be discerned surrounding the mesenchyme core (Fig 1Ac,d and 1Bc,d). The perichondrium is evident at HH32 (arrowheads, Fig 1Ae and 1Be). Note the crescent-like cap of organized otic capsule cells at the interface with the perichondrium, which is the site of the future columella footplate.

To confirm the changes in nuclei shape observed during condensation we performed quantitative analysis of nuclear circularity and calculated the nuclear deformation index, which quantifies nuclear deformation. Nuclear deformation is defined as (x-y)/(x+y), where x is the length of the major axis of a nucleus and y is the length of the minor axis of a nucleus. When the value of circularity is 1, it is a perfect circle, conversely, when NDI = 0, it is a perfect circle. Therefore, circularity and deformation index share an inverse relationship where the higher the deformation index, the lower the nuclear circularity.

We observed significant reductions in circularity in the condensed columella cells at HH30, compared to specified mesenchyme (HH24) and overtly differentiated chondrocytes (HH32, Fig 1F). Correspondingly, there was a significant increase in the nuclear deformation index during condensation, correlating with the morphological observation of stretched, rhomboid shaped cells with distorted and elongated nuclei (Fig 1F).

In summary, the formation of an organized condensation is observed at HH30, with the appearance of rhomboid-shaped cells, resulting from the synchronous transformation in cellular organization and cytoskeletal architecture. Thereafter, attaining a stable tissue conformation is a prerequisite to completion of chondrogenesis and the onset of overt chondrocyte differentiation at HH32.

### Condensation of the extracolumella is spatiotemporally distinct from the columella

Extracolumella mesenchyme is located distal/ventral to the columella mesenchyme and is bound by the lateral surface ectoderm, medial pharyngeal endoderm and dorsally situated cardinal vein (Fig 2A and 2B). The nascent perichondrium is apparent at HH30 and surrounds the extracolumella by HH31.5 (arrowheads, Fig 2A and 2B).

At HH24, the extracolumella mesenchyme cells are densely packed with irregular bundles of cortical actin (Fig 2Ca–2Ea). By HH26 this arrangement has evolved and nuclei are surrounded by dense F-actin with rigid cell-cell contacts (arrowheads, Fig 2Cb–2Eb). The nuclei display the stretched, elongated appearance we associate with condensing tissue (arrowheads, Fig 2Cb–2Eb). Thereafter, intercellular cortical actin connections remodel and between HH30 and HH31.5, where there is a reduction in the cell-cell F-actin connections, concurrent with cell-ECM focal adhesions becoming predominant (Fig 2Cc,d–2Ec,d). Continuous F-actin reorganization, between HH24 and HH31.5, changes the equilibrium of the cytoskeletal architecture, sufficient to stretch and distort the nuclei at the point of condensation (HH26), before being restored to a rounded shape during the stabilization phase (HH31.5-HH34, Fig 2C–2E). As with columella stabilization, the increasing distance between cells is due to secretion and reorganization of ECM components. At HH34, overt differentiation of chondrocytes is evident with further actin reorganization (Fig 2Ce–2Ee).

The significant loss of nuclear circularity and increase in the deformation index leads us to conclude that extracolumella condensation occurs at HH26, in contrast to HH30 in the columella.

In summary, these data indicate that the process of condensation occurs over a prolonged time period, relying on the combined influences of F-actin organization, cell-cell and cell-ECM contacts. The actual point of condensation coincides with cell nuclei being stretched and distorted from their original rounded shape, and is associated with the appearance of organized F-actin at HH26. This is followed by a period of tissue stabilization before the onset of overt differentiation of chondrocytes at HH34.

Overall, although both columella and extracolumella condensation follow the same general pattern of cell shape changes, the timing of condensation and morphology of the condensed cells are unique for columella and extracolumella. We propose that this represents a general mechanism of condensation for the cartilage elements in the head, with the difference between columella and extracolumella being attributed to dorso-ventral positional identity. We will examine this idea in greater detail in the later part of this study.

### Cell density decreases as pharyngeal mesenchyme condenses

**Fig 3:**
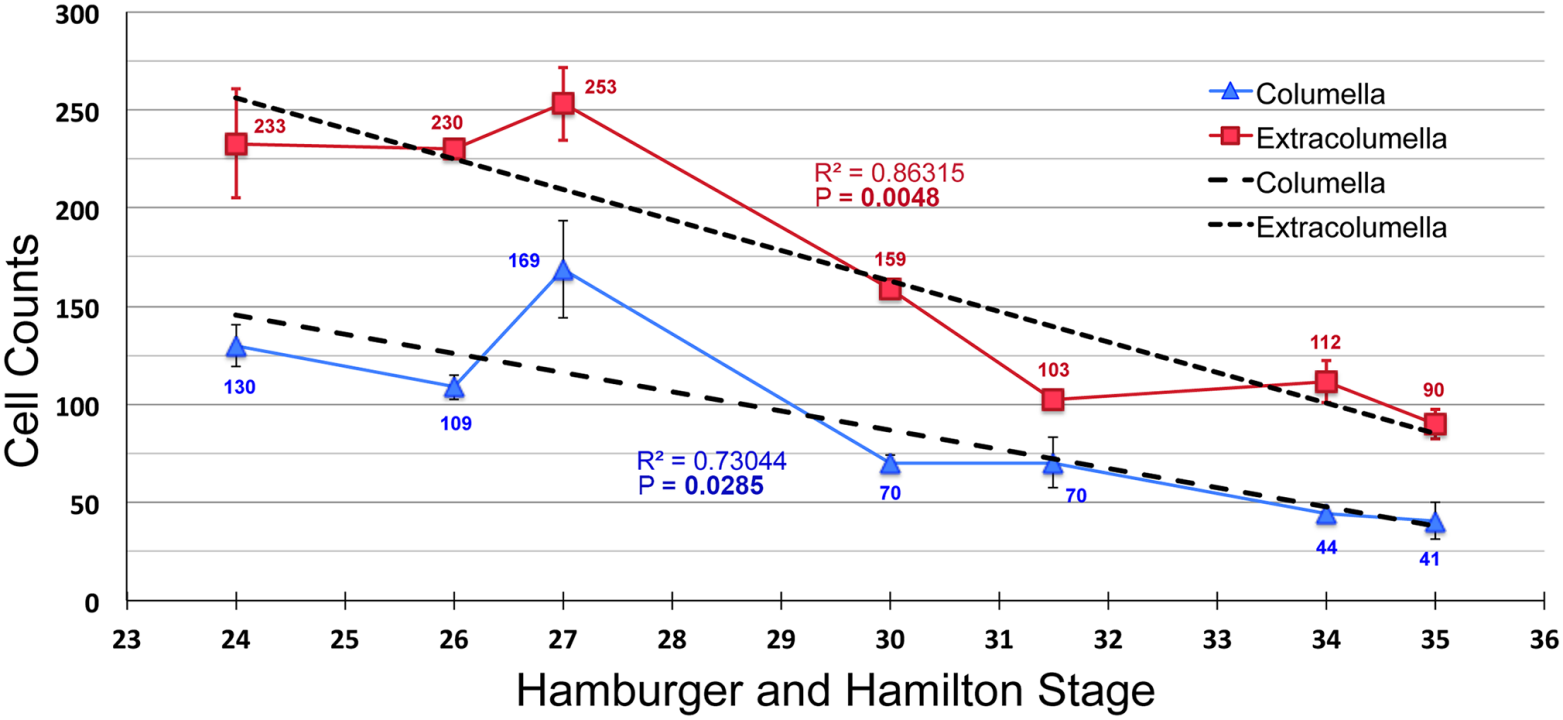
Cell density decreases during pharyngeal mesenchyme condensation. A graph of normalized cells counts showing cell density in the columella (blue) and extracolumella (red) between HH24 and HH35. The columella has lower density than the extracolumella at all time points. At HH27 there is a slight, but not statistically significant increase in cell numbers. Overall, there is a trend towards lower density over time. The rate of density reduction in the extracolumella is higher than that of the columella, R^2^ = 0.86 and 0.73, respectively. P values for the linear regression for extracolumella and columella are significant at 0.0048 and 0.0285, respectively. Bold font indicates significant P value.

Condensations are defined as an aggregation of previously dispersed cells, resulting in an increase in cell density and upon reaching a critical density these cells will condense [7]. In vivo limb condensation studies and mesenchyme stem cell condensation in micromass culture agree with this model [17]. Similar mechanisms have been assumed in the head skeleton, but surprisingly, have not previously been examined. We quantified cell density in the columella and extracolumella during condensation by counting the number of nuclei in sections from HH24-HH35 (Fig 3).

Cell numbers in the columella mesenchyme increased slightly, but not significantly, from HH24-27 during the period when no cell specific F-actin organization was observed (blue line, Fig 3). This was followed by a precipitous reduction in cell density until HH30 when condensation occurs. Thereafter, between HH30-35, the rate of reduction was more gradual. A similar trend is observed in extracolumella mesenchyme (red line, Fig 3), which had higher cell density than the columella at all time points measured. At HH27, cell density was at its peak, as it was for the columella. Thereafter, density decreased even more markedly until HH31.5, after which the tissue stabilized. Even as overt differentiation commenced at HH34, the extracolumella was over twice as cell dense as the columella. By measuring the average slope, the rate of declining cell density was 0.86 for the extracolumella, whereas the columella had a lower overall rate of 0.73. The p values for the linear regression of cell density decline for columella and extracolumella were 0.0285 and 0.0048 respectively, both statistically significant.

Growth of the embryonic head is occurring rapidly during these stages. Several mechanisms likely facilitate the expansion of the nascent cartilage. EdU labeling showed little to no cell proliferation that could account for the increase in size (data not shown). TUNEL labeling revealed that cell death levels were also non-detectable (data not shown). We propose that the cell shape changes occurring during condensation, together with ECM deposition and ECM realignment [15, 16, 18] act together to push cells away from the center, resulting in tissue expansion and a reduction in cell density. Similar tissue reorganization occurs in the tooth mesenchyme [4, 19]. Additionally, the adjacent tissues surrounding the columella and extracolumella may additionally ‘pull’ the mesenchyme tissue, aiding its expansion.

In summary, cell shape changes and cytoskeletal re-organization are highly informative measures when defining the spatiotemporal aspects of condensation. These measures may prove more informative than the classical condensation definition (an aggregation of cells and an increased/critical cell density), in the pharyngeal and other non-limb condensations. Next, we next tested the requirement for actin based cell shape changes and cytoskeletal reorganization.

### Cytoskeletal reorganization of F-actin is required for condensation and cell shape changes

Middle ear mesenchyme specification in the columella occurs at HH18, condensation at HH30 and overt differentiation at HH32-34 [1, 12]. After HH24, multiple membranes make the embryonic head and pharyngeal arches inaccessible in ovo. To access the pharyngeal arches between specification and overt differentiation we developed an in toto slice culture system. A transverse slice from the chick head, encompassing the brain and the entire second pharyngeal arch region, was extirpated and cultured flat on a porous membrane (see materials and methods). This arrangement preserved the physical integrity and molecular signaling characteristics of the tissue.

**Fig 4:**
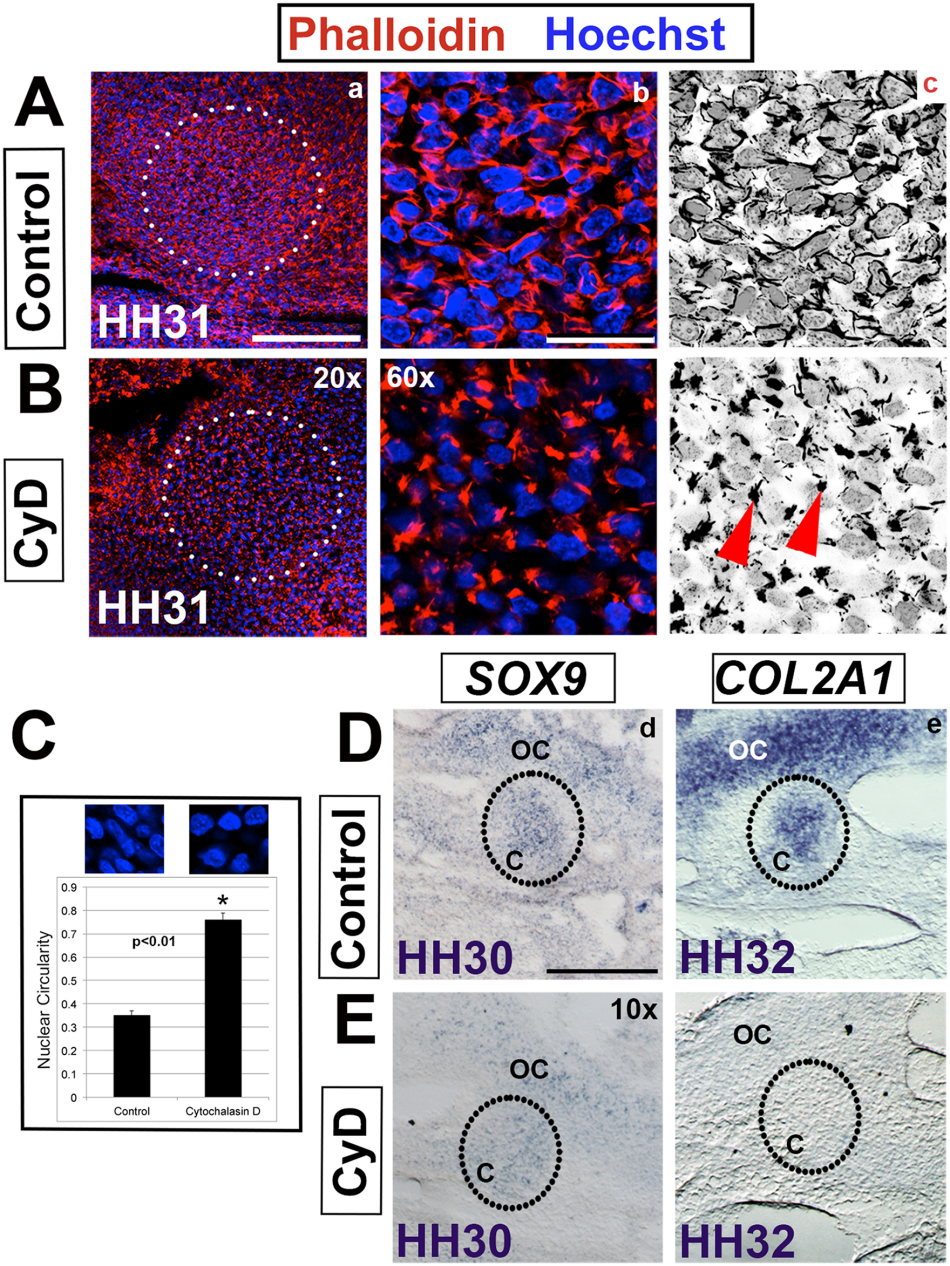
Cytochalasin D prevents cytoskeleton rearrangement and condensation. (A,B) The columella region (circled) at HH31 showing control and Cytochalasin D (CyD) treated tissue. (a,b) Phalloidin (red)–actin, Hoechst (blue)—nuclear DNA. (c) Black and white view to highlight nuclei and actin. (A,B) Inhibition of actin polymerization results in loss of cell-cell connections (b,c). Rather, a clump of actin (arrowheads) is observed at one pole of the cell. (C) Nuclear circularity is normally reduced during the process of condensation (controls) at HH30. In Cytochalasin D treated tissue the nuclei maintain their pre-condensation rounded shape, indicating failure of condensation (asterisk, p<0.01). (D,E) In situ hybridization of chondrogenesis markers in control and Cytochalasin D treated tissue. (d) *SOX9* expression, as an indicator of ongoing chondrogenesis, is reduced at HH30 and (e) *COL2A1* fails to be induced in treated explants at HH32 indicating a failure of overt differentiation. Abbreviations: c-columella, oc- otic capsule. Scale bars represent 150 μm (10x), 75 μm (20x), and 25 μm (60x).

To test the requirement for actin based cell shape changes and cytoskeletal reorganization we applied 50 μM of Cytochalasin D (an inhibitor of actin polymerization) in the growth medium at HH28, a day before the appearance of the long intercellular processes and rhomboid cell shapes that characterize condensation (HH30) [20, 21]. Analysis was performed at HH31 to ensure that there was not just a delay in condensation. Condensation occurred as expected in control slices (n = 3) and was identical to sections from intact heads at corresponding stages (Fig 4Aa–4Ac and not shown). In contrast, in treated explants (n = 4) the tissue was disorganized (Fig 4Ba) and actin was polarized at one end of the cell (arrowheads) and the actin bridges (cell-cell connections) were absent (Fig 4Bb and 4Bc). Additionally, analysis of nuclear circularity at HH30 showed that nuclei remained significantly rounded in treated explants, rather than deforming as expected during condensation (Fig 4C). The failure of the nuclei to deform from a rounded, pre-condensation shape indicates that condensation failed to occur.

We proposed that disrupting actin polymerization affects the pulling force generated by intercellular cell-cell adhesion and the traction forces of cell-ECM connections. The loss of opposing forces forestalled condensation, as indicated by the failure to achieve the rhomboid cell shapes and stretched nuclei observed in control tissue. Additionally, inhibiting polymerization disturbs the tension resistant forces produced by the interaction of cortical actin with the internal cytoskeleton interfering with tissue stabilization [3, 22].

In summary, these data support a model of actin-based cytoskeletal rearrangements and associated cell shape changes that are required to establish the differential tensile forces (cell-cell, cells-ECM and internal cytoskeletal) driving condensation. We next sought to determine if condensation and stabilization are required for chondrocyte cell fate.

### Condensation is required for overt differentiation into chondrocytes

*SOX9* and *COL2A1* are markers of pre-chondrogenic and chondrogenic identity, respectively [1, 12]. In situ hybridization expression analysis detected *SOX9* transcripts in specified chondrocytes in the columella and the otic capsule at HH30. In Cytochalasin D treated explants, however, reduced levels of *SOX9* transcripts were detected (Fig 4Dd and 4Ed). This suggests that specified pre-chondrogenic cell fate [1, 12] is partially disrupted in the absence of cytoskeletal reorganization. *COL2A1* expression was induced as normal in controls at HH32, indicating overt differentiation (Fig 4De). In contrast, Cytochalasin D treated explants failed to express *COL2A1* in either the columella or otic capsule (Fig 4Ee).

These data support a model in which condensation is required for chondrogenesis. *SOX9* directly regulates *COL2A1* expression and is essential for overt differentiation of chondrocytes [23, 24]. Continued down regulation of *SOX9* after HH30 is predicted, although we have not explicitly tested this. Cytochalasin D treated explants fail to undergo overt differentiation as evidence by the failure to induce of *COL2A1* expression. Given that interfering with actin polymerization affected not just cell shape, but also gene expression, we next determined the effect of inhibiting actin polymerization on the BMP, FGF and TGF-β signaling pathways, which also have roles in condensation and chondrogenesis.

### Cytoskeletal reorganization modulates activated FGF, BMP and TGF-β signaling

Cytoskeletal reorganization potentiates cell shape changes and importantly, this reorganization modulates mechanotransduction between the cell membrane and the nucleus, directly affecting downstream gene expression and thus, cell fate switching [3–5]. Cell shape and cell tension influence lineage choice between osteocytes and adipocytes [5]. Additionally, using tooth mesenchyme, Mammoto and colleagues showed that cytoskeletal reorganization modulates the levels of transcription factors within tissue [4]. Several groups have postulated that during endochondral ossification the physical forces remodeling cortical actin and cell shape influence molecular signaling pathways [25–27].

We tested the effect of interfering with cytoskeletal rearrangements and associated tensile forces on select signaling pathways. We monitored the effect of actin disruption using antibodies against the downstream mediators of the BMP, FGF and TGF-β signaling pathways: pSmad1/5/8, pERK and pSmad2, respectively [28]. Although part of the same superfamily, BMP and TGF-β often exhibit antagonistic activity, regulating activation of different downstream Smads; R-Smads and Smad2/3, respectively [29]. Phosphorylated Smads translocate to the nucleus and interact with transcription factors that regulate gene expression of factors that promote cell fate decisions [30, 31].

**Fig 5:**
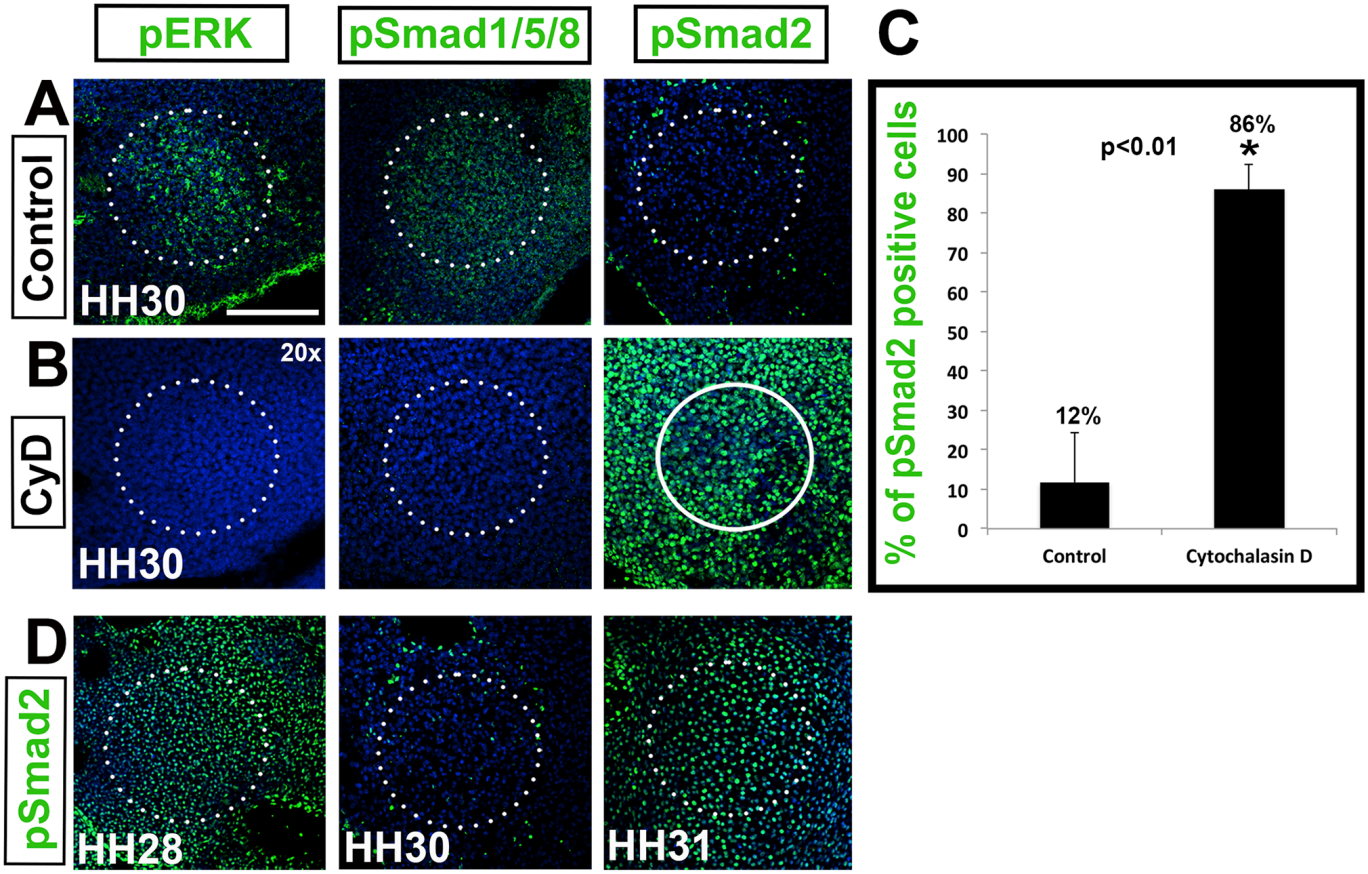
Actin reorganization is required for columella condensation. (A,B) Tissue slices treated with Cytochalasin D. The activation status of FGF, BMP and TGF-β signaling pathways in the columella (circled) as measured by pERK, pSmad1/5/8 and pSmad2 (green) immunolabeling, respectively, at HH30. (B) Cytochalasin D treatment reverses the activated signaling patterns. (C) Graph of pSmad2 positive cells at HH30. Compared to 12% of control cells, 86% of cells remained pSmad2 positive in treated explants (asterisk, p<0.01). (D) TGF-β signaling in control tissue, showing the normal expression of TGF-β between HH28 and HH31. pSmad2 immunolabeling shows that TGF-β signaling is high at HH28 before condensation, down regulated at HH30 during condensation, and up regulated at HH31 following condensation. Scale bar represents 75 μm (20x).

In controls (Fig 5A), activated BMP and FGF signaling were detected at HH30, as measured by the presence of pSmad1/5/8 (n = 3) and pERK (n = 3), respectively, whereas no Smad2 signaling was detected (n = 5/ Fig 3E). Satisfyingly, the reverse outcome was observed in Cytochalasin D treated explants (Fig 5B) where pSMAD1/5/8 (n = 3) and pERK (n = 3) were not detected, whereas pSmad2 was highly up regulated, indicating active TGF-β signaling (n = 5). In normalized samples, 86% of cells were pSmad2 positive, compared to 12% of control cells (Fig 5C). Samples were normalized by counting the number of labeled cells versus the total number of cells in a set area (see materials and methods). This result is significant for two reasons. Firstly, it demonstrates that actin polymerization and associated cytoskeletal reorganization are sufficient to influence molecular signaling, and secondly, that the effects are not indiscriminate, but pathway specific.

Interestingly, when we observed pSmad2 labeling over a number of stages it became apparent that the down regulation TGF-β signaling was a transient phenomenon, occurring at HH30 –at the point of columella condensation (Fig 5D). At HH28, high levels of pSmad2 are detected in the putative columella condensation, followed by temporary down regulation during cytoskeletal reorganization and formation of rhomboid cell shapes at HH30, when we define condensation as occurring (see Fig 1Cc). One stage later, the level of active pSmad2 is restored. In comparison, Cytochalasin D treated tissue maintained active TGF-β signaling at all the stages tested, including HH30, in the absence of dynamic cytoskeletal reorganization (n = 5, Fig 5D).

A mechanical continuum linking cell-cell and cell-ECM interactions at the plasma membrane is transmitted to the nucleus directly modulating downstream gene expression [3, 31]. Similarly, shear stress induced cell shape changes and genetic analysis show that gene expression is altered in response to the duration and magnitude of mechanical force applied [30]. Our data support this model of mechanotransduction, linking the internal cytoskeleton via cortical actin and microtubules, connecting the cell membrane with the nucleus of condensing cells, that then results in transient down regulation of pSmad2 [4, 30, 31].

In summary, we show that interfering with actin polymerization and associated tensile forces is sufficient to alter the phosphorylation status of downstream effectors. It is important to note that changes in the phosphorylation status of Smad1/5/8, ERK and Smad2 are the results of cytoskeletal reorganization, not the cause. We propose that differential tensile forces positively regulate BMP and FGF signaling, and negatively regulate TGF-β signaling during columella condensation. This is significant because it supports the hypothesis that physical forces have a direct influence on cell fate decisions though the mechanical continuum with the nucleus [4, 30, 31].

### Differential cell cortex tension and actomyosin contractions regulate condensation

**Fig 6:**
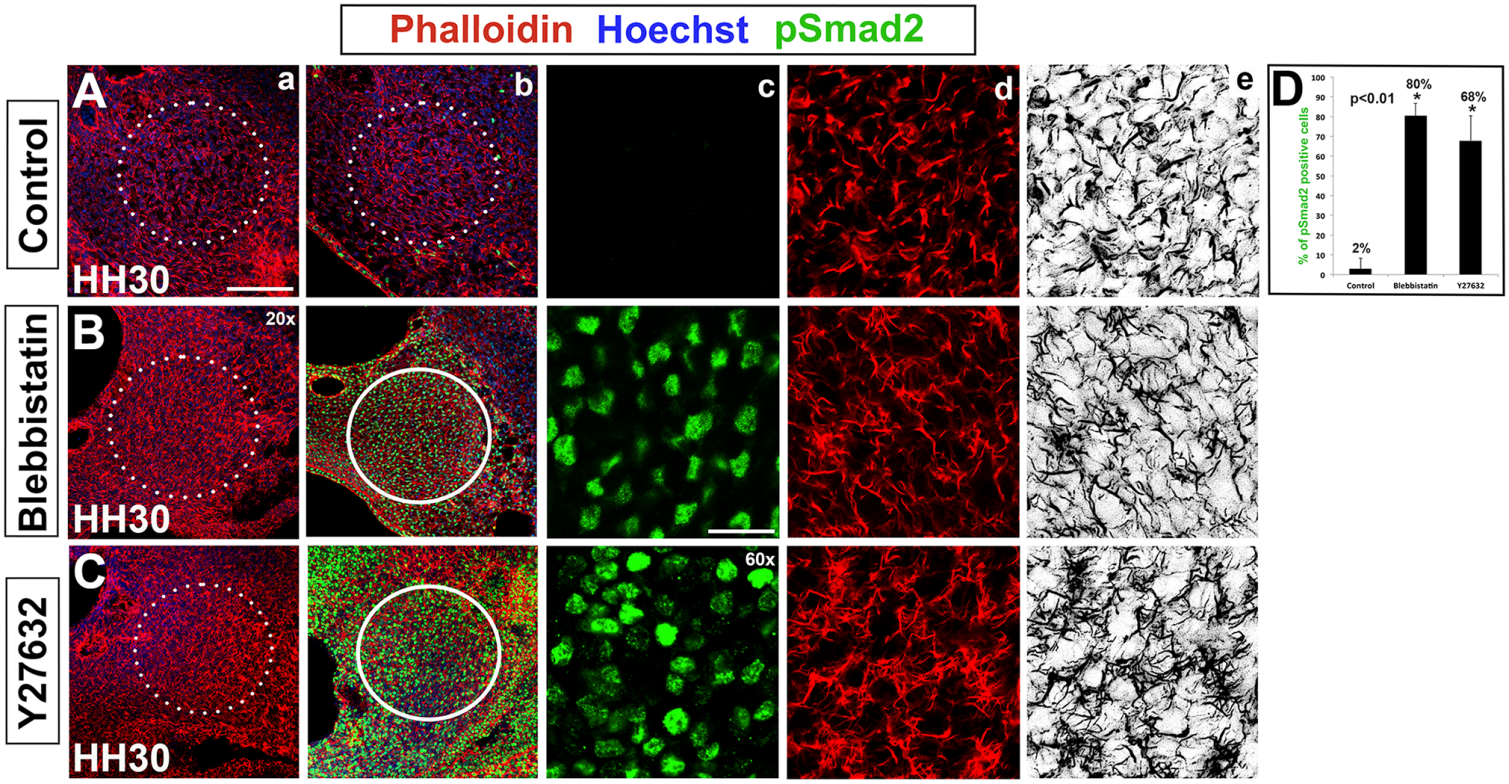
Myosin II and ROCK inhibition disrupts columella condensation. (A-C) Tissue slices treated with Blebbistatin (Myosin II inhibitor) or Y27632 (ROCK inhibitor). (a-d) Hoechst (blue)—nuclear DNA, Phalloidin (red)—F-actin, pSmad2 (green)—TGF-β signaling. (e) Black and white view of F-actin. (A) Normal condensation in controls with pSmad2 down regulated at HH30 (c) and formation of cell-cell actin bridges (d,e). (B,C) TGF-β signaling is maintained in explants exposed to Blebbistatin or Y27632 as indicated by high levels of pSmad2 immunolabeling (c). Condensation doesn’t occur in treated explants as indicated by the lack of an organized tissue (a) and disorganized actin (d,e). (D) Quantitative analysis indicates 2% of pSmad2 positive cells in controls, whereas pSmad2 is maintained in 80% of Blebbistatin treated cells and 68% of Y28632 treated cells (asterisk, p<0.01). Scale bars represent 75 μm (20x) and 25 μm (60x).

To test the role of differential cell cortex tension in driving mesenchyme condensation we targeted ROCK and Myosin II (Fig 6). ROCK regulates actomyosin contractions and actin filament dynamics [3]. Myosin II has a role in mediating the actomyosin-dependent cell-cortex tension [22]. Intercellular surface tension results from the interaction of cortical tension and adhesion. The inward-pulling actomyosin contractile network functions to minimize the contact surface between cells and is opposed by cell-cell and cell-ECM adhesive forces that work to increase surface contact. Changes in the equilibrium between these forces introduce differential tension, which we hypothesize drives dynamic cytoskeleton rearrangements and concomitant cell shape changes.

Firstly, we inhibited Myosin II function with Blebbistatin, which exhibits high affinity binding against the myosin-ADP-Pi complex and interferes with phosphate release, resulting in Myosin becoming locked into a low affinity conformation against actin [32]. We predict that interfering with the rigid actomyosin cross-linking that serves to establish differential cortex tension [22] will render condensation impossible. TGF-β signaling is normally transiently suspended at HH30 with the onset of condensation (n = 4, Fig 6A). The loss of differential cortex tension upon application of Blebbistatin at HH28 results in maintenance of Smad2 phosphorylation (80%) and continued TGF-β signaling at HH30 (Fig 6B and 6D). The long actin processes and rhomboid cell shapes characteristic of condensation fail to form (n = 5). Rather, actin is arranged loosely in the same disorganized manner seen in pre-condensation mesenchyme (compare with Fig 1Cb).

RhoA/ROCK signaling is well characterized as a regulator of cell shape, regulating the cytoskeleton through actomyosin contraction in a context dependent manner [33–35]. In vitro experiments disrupting ROCK (Rho associated protein kinase) and RhoA (RhoA-GTPase) activity lead to failure of actomyosin contraction based cell shape changes, directly influencing cell fate choice, but to date this has only been demonstrated during chondrocyte differentiation, not during condensation [34–36]. Additionally, ROCK can mediate downstream expression of TGF-β signaling [36], and TGF-β related signaling in turn modulates actomyosin-dependent cell-cortex tension of germ-layer progenitors [22]. This suggests that in pharyngeal mesenchyme the process of condensation can be influenced by interfering with ROCK mediated actomyosin contractions.

ROCK function was inhibited to investigate whether Rho Kinase mediated signaling is required for cell shape changes and more specifically, the establishment of the long actin protrusions observed at HH30 in the columella condensation (Fig 6C). Indeed, TGF-β signaling is maintained when ROCK is inhibited by application of 250 μM of Y27632 (68%, n = 5, Fig 6C and 6D). Furthermore, cell shapes remain disorganized and fail to achieve the rhomboid, stretched shape observed at condensation.

In summary, Myosin II and ROCK inhibition, in addition to our Cytochalasin D experiments, demonstrates that modulating cell-cortex tension results in maintenance of TGF-β signaling at HH30, an indication of failure of the mesenchyme to condensate. Moreover, phalloidin labeling shows that cell processes maintain the numerous actin fibers and irregular cell protrusions characteristic of pre-condensation morphology seen at HH28. This is significant because it implies that Myosin II and ROCK mediated actomyosin contraction and cell-cortex tension are required to induce differential tension that drives condensation.

### Inhibition of FGF, BMP and TGF-β signaling does not affect condensation or chondrogenesis

**Fig 7:**
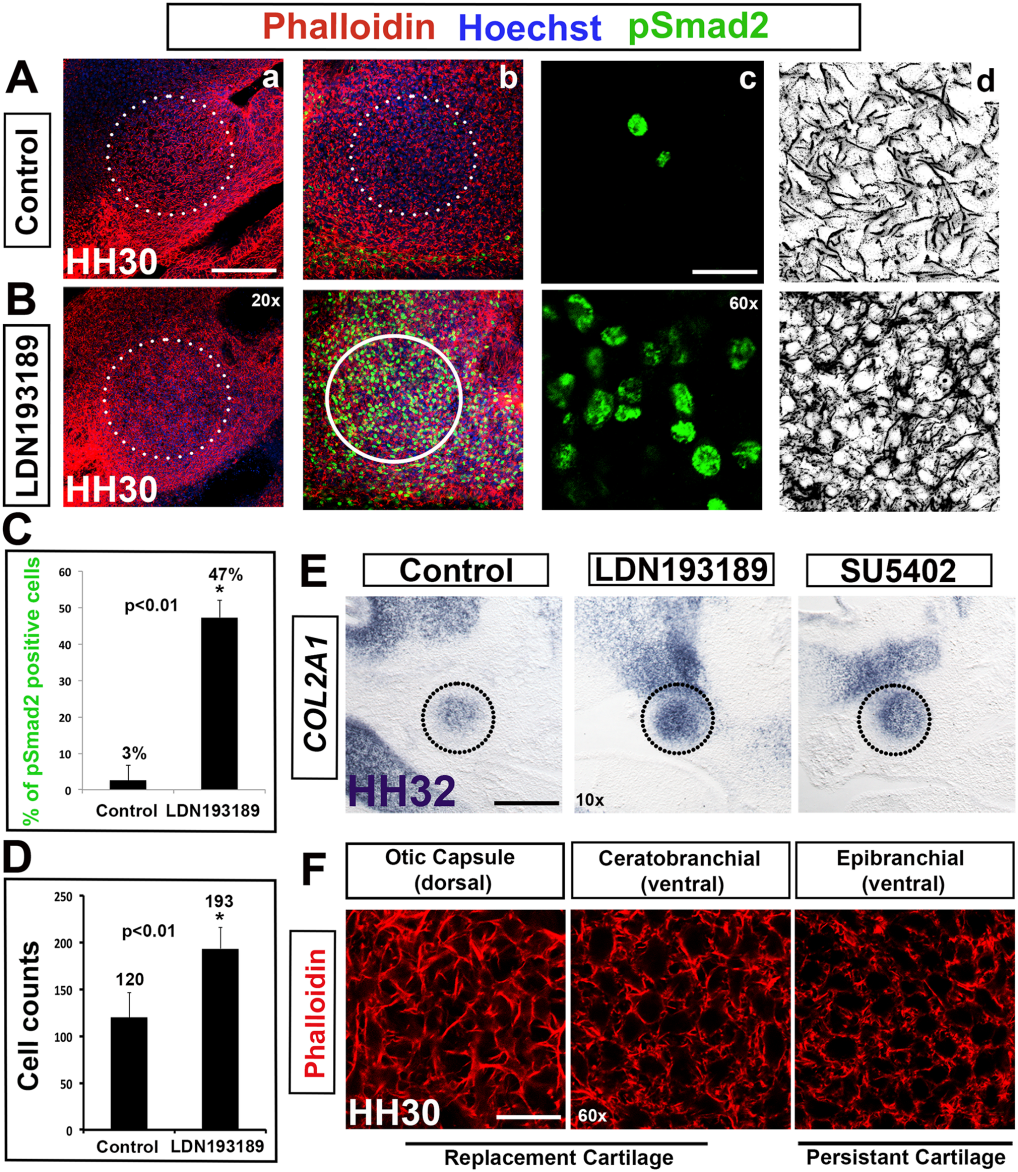
BMP inhibition results in dorsal to ventral transformation of columella condensation morphology. (A,B) Application of the BMP inhibitor LDN193189. (a-c) Hoechst (blue)—nuclear DNA, Phalloidin (red)—F-actin, pSmad2 (green)—TGF-β signaling. (d) Black and white view of F-actin. (C,D) Graphs comparing percentage of pSmad2 positive cells and cell density in controls and LDN193189 treated tissue. (E) *COL2A1* in situ hybridization in LDN193189 and SU5402 treated tissue. (F) Phalloidin immunolabeling of dorsal and ventral cartilages of the second pharyngeal arch. (A,C) pSmad2 is detected at HH30 in 3% of controls. (B,C) Where BMP is inhibited, TGF-β signaling is maintained in a significant number of cells (47%, asterisk, p<0.01). (d) Tissue exposed to LDN193189 exhibits morphology that is reminiscent of the ventrally situated extracolumella. (D) Similar to extracolumella cell counts, LDN193189 treated tissue has an increased cell density compared to controls (asterisk, p<0.01). (E) *COL2A1* expression is unaffected by the inhibitors LDN193189 (BMP) and SU5402 (FGF) indicating no effect on overt differentiation of chondrocytes. (F) Dorsal (otic capsule) and ventral (ceratorbranchial and epibranchial) cartilage have distinct actin morphology, irrespective of their fate as replacement or persistent cartilage. Scale bars represent 150 μm (10x), 75 μm (20x) and 25 μm (60x).

BMP signaling regulates chondrogenesis at multiple stages [10, 37–39]. Combined BMP and FGF signaling is required early to specify prechondrogenic identity [1]. WNT signaling has a well-established role in limb chondrogenesis and is required for osteogenesis [40]. To establish the role of these signaling pathways in pharyngeal condensation, we applied BMP, FGF (SU5402) and WNT (XAV939) pathway inhibitors to our in toto explant cultures (Fig 7).

LDN193189 prevents Smad1/5/8 phosphorylation by inhibiting the BMP type I receptors, ALK2 and ALK3 [41]. SU5402 targets FGF receptor tyrosine kinase activity, and XAV939 inhibits the WNT pathway by stabilizing AXIN through inhibition of the poly-ADP-ribosylating enzymes tankyrase 1 and tankyrase 2, resulting in beta-catenin degradation through the ubiquitin-proteasome pathway [42]. Application of 100 μM LDN193189 (n = 3), 100 μM SU5402 (n = 3) or 200 μM XAV939 (n = 3) at HH28 had no effect on *COL2A1* expression at HH32. These data indicate that chondrocytes underwent condensation and overt differentiation, unaffected by down regulation of BMP, FGF (Fig 7E) and WNT signaling (not shown).

### BMP signaling is instructive for dorsal morphology in cranial condensations

BMP inhibition, however, did affect levels of TGF-β signaling, cell density during condensation and condensation morphology (Fig 7A and 7B). pSmad2 is normally transiently downregulated in the dorsal columella condensation at HH30 (Fig 7A) at the point of condensation. In controls, only 3% of cells maintain pSmad2 at HH30. With the suppression of BMP signaling 47% of cells maintain pSmad2 (n = 15) (graph, Fig 7C), demonstrating the antagonistic relationship between these two pathways. Additionally, cell density was maintained at higher levels rather than being reduced by almost half as occurred in control condensations (193 vs. 120), (graph, Fig 7D and blue line, Fig 2).

Closer examination of the morphology suggested a surprising possibility: that the columella condensation had adopted a ventral morphology. The LDN193189 treated columella displayed a packed polygonal morphology and tight cell-cell connections (black and white image, Fig 7B), reminiscent of the ventral extracolumella condensation at HH26 (Fig 2Ca). Similarly, the dorsally located otic capsule condensation, that has a heterogeneous origin composed of neural crest, cranial mesoderm and first somite mesoderm [43] had also adopted the more packed ventral-like morphology in the absence of BMP signaling. Thus, we hypothesize that in the absence of instructive BMP signaling that the dorsal condensation shifts to a ventral condensation program, and indeed that this might be the ground state of pharyngeal condensations.

Notably, although the BMP inhibitor was applied globally to the in toto explant, the ventral extracolumella showed no changes in morphology. The ventral extracolumella condensation cells do not normally experience BMP signaling (indicated by the lack of phosphorylated Smad1/5/8). The extracolumella also normally exhibits negligible levels of Smad2 phosphorylation. Addition of recombinant BMP protein had no influence on the morphology of the dorsal or ventral condensation, indicating that the activation status downstream of the ligands is critical. Together these data indicate that the ventral condensation morphology is BMP-independent and indeed, is refractive to application of the BMP inhibitor.

We also added SB43152, a selective inhibitor of TGF-β signaling along with LDN193189 (BMP inhibitor) to determine whether inhibiting TGF-β signaling rescues the effect of BMP inhibitor on the dorsal columella condensation. SB43152 inhibits TGF-β signaling mediated activation of SMAD proteins by blocking TGF-β superfamily type I activin receptor-like kinase (ALK5, ALK4 and ALK7) receptors [44]. Addition of SB43152 indeed rescued the effect of the BMP inhibitor. The columella condensation formed normally with no ventral transformation evident using Phalloidin and Hoechst staining (not shown). This result suggests that BMP signaling imparts dorsal condensation morphology by transient downregulation of TGF-β signaling.

The columella is formed from replacement cartilage, becoming ossified beginning at E13.5, whereas the extracolumella cartilage persists throughout life [12]. Could the above result be related to these cell fates? We examined several second arch condensations and found that persistent or replacement cartilage fates had no bearing on the differential cell shapes between dorsal and ventral (Fig 7F). Moreover, the cellular origin of each element had no bearing on the effect of BMP inhibition, for example, the multiple origins of the otic capsule tissue and neural crest derived cartilage. The only difference we established was the cartilages from proximal/dorsal mesenchyme had the distinct dorsal morphology of the columella, whereas distal/ventral cartilages looked more like the extracolumella.

In summary, these data indicate that dorsal skeletal mesenchyme, regardless of cellular origin, or fate (as persistent/replacement cartilage) requires instructive BMP signaling to adopt a dorsal morphology, whereas the more ventral mesenchyme situated proximally within the arches is BMP-independent. Thus, our results suggest that in the absence of an instructive BMP signal within dorsal condensations such as the columella condensation, dorsal condensations will adopt ventral morphology.

## Discussion

Tissue organization during development exhibits coordinated cell behaviors involving tensile forces, differential cell adhesion and actomyosin based cell cortex tension, which regulate multiple morphogenetic processes [45]: germ layer organization [46], convergent extension during gastrulation [47–49], drosophila germ band extension [48, 50] and dorsal closure [46]. Modulation of the mechanical equilibrium drives cytoskeletal reorganization, alters the mechanical continuum between the cell membrane and nucleus, nuclear shape and polarization [31, 51], which in turn regulates downstream molecular signaling and gene expression [3, 52].

Our results identify ROCK-mediated actomyosin contractions together with ROCK-dependent Myosin II generated differential cell cortex tension as essential generators of cytoskeletal reorganization. ROCK inhibition produces no obvious consequences on limb [7, 53, 54], tooth [4, 19] or kidney condensations [55]. Unexpectedly, we observed a stretching effect, producing rhomboid shaped cells and deformed nucleus, at the culmination of condensation. This mimics RhoA function during morphogenesis, but is not observed during limb and kidney condensation, which require suppression of RhoA function [33–35, 55]. This supports the self-organization model in the specified mesenchyme of pharyngeal arches in contrast to the tooth and kidney condensation model, which is driven by epithelium-derived compressive forces. In agreement with data demonstrating mechanical push and pull forces in tooth condensation [4, 19], we propose that all skeletal condensations require tensile forces during their morphogenesis, a rich avenue for future research. Thus, this study suggests that ROCK signaling has a unique role in pharyngeal arch-derived cartilage.

The definition of condensation conjures images of cell compaction driven by compressive forces. Our results note two major deviations from this standard model; firstly, cell membranes that are stretched during condensation, producing rhomboid shaped cells and stretched nucleus, and secondly, the lowering of cell density in the pharyngeal dorsal and ventral condensations. Both effects are the results of ROCK/Myosin II induced cell shape changes, adaptive focal adhesions and ongoing production of ECM deposits, changes in ECM composition and realignment of ECM components [13, 14, 54, 56]. ROCK/Myosin II-dependent assembly of cortical actin promotes filopodia-like cell-to-cell connections and the characteristic rhomboid cell shape changes required for mesenchymal condensation, the gateway to chondrogenesis [56].

Remarkably, manipulating the polymerizing activity of cortical actin modulates the downstream molecular response of BMP, FGF and TGF-β signaling. In contrast, manipulating these signaling pathways had no impact on the ability of the mesenchyme cells to undergo condensation. Moreover, although we show that BMP signaling is irrelevant for the process of condensation, it is required for dorsal morphology, indicating that condensation and dorsal cell shape can be uncoupled. The loss of BMP signaling results in ventral condensation characteristics suggesting that the BMP-independent ventral pattern is the ground state for pharyngeal cartilage. FGF signaling from pharyngeal endoderm and the surface ectoderm of the arches during earlier specification [1] is a potential mediator of this base state. Moreover, ventral condensations are refractory to BMP signaling, supporting the hypothesis that the ground state in pharyngeal condensations is ventral. Additionally, an antagonistic relationship exists between BMP and TGF-β signaling. BMP signaling promotes chondrocyte differentiation and cell proliferation, while down regulating TGF-β signaling, thus protecting cells from TGF-β’s repressive effects [29].

It appears that the level of Smad2 phosphorylation acts as a readout of condensation in the pharyngeal arches: 2–3% of pSmad2 positive cells occur in a normal dorsal condensation; 47% of pSmad2 positive cells in tissue with BMP inhibition that results in a dorsal to ventral transformation; and in cases of failure of condensation in tissue treated with cytoskeletal inhibitors there are 68–86% Smad2 positive cells. The percentage of pSmad2 expressing cells is significantly different (p value <0.05) between the ventrally transformed condensation (BMP inhibitor—LDN193189) and in cases of failure of condensation (Cytochalasin D, Blebbistatin and Y27632). Thus, in the absence of negative regulation by BMP, the dorsal mesenchyme has an inherent predisposition to maintain TGF-β signaling, a property unique to the dorsal cells.

These data settle an open question on the role of BMP signaling in condensation and overt differentiation. It was proposed that BMP signaling was required for condensation, however addition of BMP2 to a culture of chondrogenic cells prevented condensation, with overt differentiation still occurring in these experiments suggesting that condensation and chondrogenesis could be uncoupled [57]. Our results provide evidence to refute this notion. We show that condensation is essential for determining chondrogenic fate and thus, overt differentiation. However, this is the result of physical forces that impart tensile stresses on the actin cytoskeleton, changing the shape of the nucleus and cell fate, whereas BMP is required for regional morphology, i.e. dorsal identity, rather than condensation. Thus, BMP is inherently uncoupled from condensation and overt differentiation. Instead, condensation is driven by mechanical forces that are essential for determining chondrocyte fate and thus, overt differentiation.

What then, is the significance of BMP induced differential dorsoventral patterning in pharyngeal condensations? BMP acts in an instructive capacity by regulating differential cell cortex tension, which leads to the observed cell shape changes and most likely plays a crucial role in cell fate specification from a prechondrogenic identify toward overt differentiation. Moreover, the stretching of dorsal mesenchyme suggests that they are subject to greater differential cell cortex tension than the more compact, ventral, BMP-independent cells. Perhaps a threshold of differential cell cortex tension is required for cartilage condensation and transient downregulation of TGF-β signaling. Above a certain threshold, condensations adopt either dorsal or ventral morphology with the former having higher levels of differential cell cortex tension due to active BMP signaling. Others have demonstrated that these cytoskeletal dependent physical forces lead to deformation of the internal cytoskeleton and organelles, especially the nucleus [31]. This could explain the down regulation of active TGF-β signaling [3, 30, 31]. Moreover, BMP signaling has a known role in condensation size and shape. Darwin finches with short/broad beaks have higher, early levels of BMP exposure than finches with long/sharp beaks. This morphological divergence is established during very early stages of morphogenesis [38, 58]. In the chicken, dorsal condensations—BMP-exposed dorsal tissue—results in broad/flat cartilages (columella, quadrate), whereas BMP-independent ventral cartilages (extracolumella, epibranchial, ceratobranchial) are elongated and thin [59]. The cytoskeletal geometry and spatial coordinates of external boundaries define cell shape. Balanced intrinsic and extrinsic mechanical forces on the plasma membrane maintains cell shape and determines the size and shape of tissues, furthermore acting in regulating cell fate decisions [3, 16, 52, 60, 61]. Changes in tensile forces during condensation, mediated by BMP exposure and TGF-β regulation, dictate cytoskeletal rearrangements in dorsal condensations and in turn, actin geometry determines the orientation of cell division, directing the direction of growth [62]. These data suggest an integrated mechanism of mechanical forces and molecular signaling is initiated during condensation that determines cartilage shape and size.

**Fig 8:**
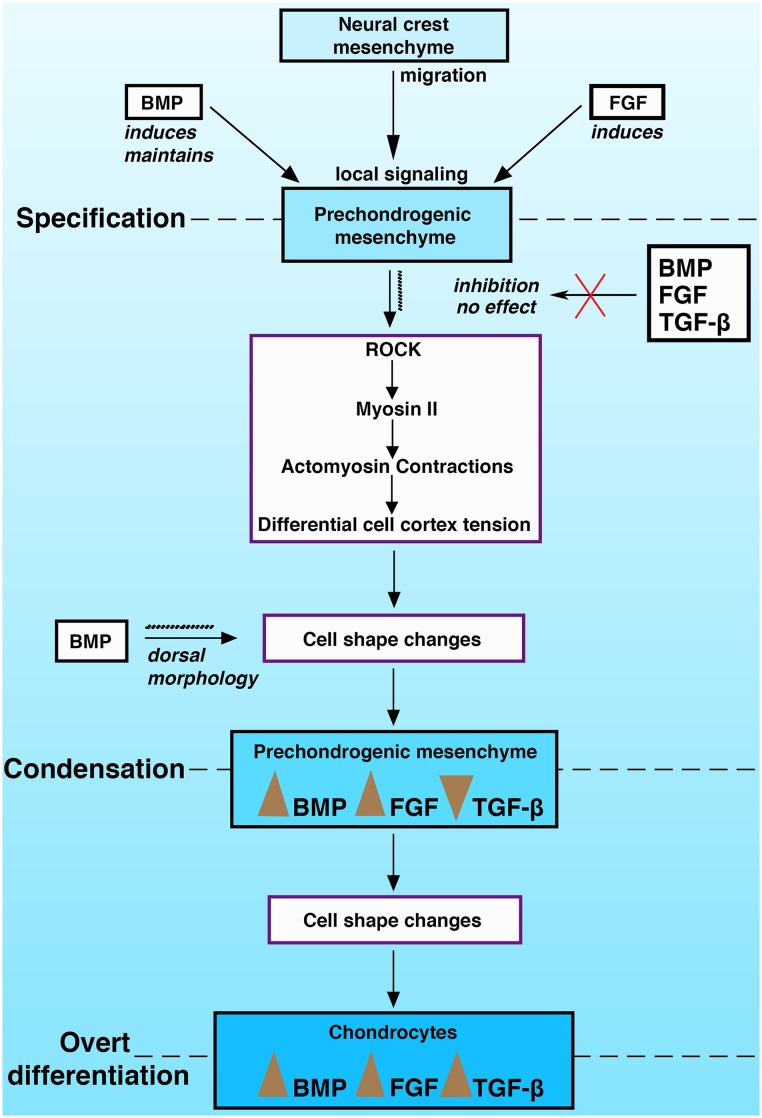
Model of skeletal condensation. A framework of the steps during endochondral ossification in the pharyngeal arch skeletal elements during specification, condensation and overt differentiation.

This study together with our previous findings [1] suggests a cascade of interrelated steps necessary for endochondral ossification in the pharyngeal arches (Fig 8). Local BMP and FGF signaling induce prechondrogenic cell fate in neural crest mesenchyme that has migrated to the pharyngeal arches. BMP is required to maintain this fate, which in turn promotes ROCK/Myosin II mediated self-assembly of the mesenchyme, through a combination of tensile force driving cell shape changes. Actomyosin contractions and differential cell cortex tension lead to cell shape changes and the physically mediated regulation of downstream signaling activity of BMP, FGF and reduced TGF-β signaling. These forces are responsible for the size and shape of the condensation, stimulating the nascent cartilage cells to undergo overt differentiation into chondrocytes, which in turn exhibit enhanced BMP, FGF and TGF-β signaling [61]. Notably, TGF-β activation of Smad2/3 specifically activates RhoA/ROCK mediated cytoskeletal reorganization and chondrocyte specific gene expression in mesenchyme stem cells [36]. This suggests that at the point of condensation TGF-β activity must be suppressed in order to maintain prechondrogenic identity, but thereafter, active TGF-β signaling induces overt differentiation into chondrocytes through Smad2/3 mediated signaling.
